# Cytotoxic activity of the twigs of *Cinnamomum cassia* through the suppression of cell proliferation and the induction of apoptosis in human colorectal cancer cells

**DOI:** 10.1186/s12906-018-2096-x

**Published:** 2018-01-25

**Authors:** Gwang Hun Park, Hun Min Song, Su Bin Park, Ho-Jun Son, Yurry Um, Hyun-Seok Kim, Jin Boo Jeong

**Affiliations:** 1 Forest Medicinal Resources Research Center, National Institute of Forest Science, Yeongju, 36040 Republic of Korea; 2 Department of Medicinal Plant Resources, Andong National University, Andong, 36729 Republic of Korea; 3 Department of Food Science & Biotechnology, Kyonggi University, Suwon, 16227 Republic of Korea; 4 Insititute of Agricultural Science and Technology, Andong National University, Andong, 36729 Republic of Korea

**Keywords:** Anticancer, ATF3, *Cinnamomum cassia*, Cyclin D1;.Human colorectal cancer, NF-κB, ROS, Twigs of *Cinnamomum cassia*

## Abstract

**Background:**

Because twigs of *Cinnamomum cassia* (TC) have been reported to exert anti-cancer activity, the mechanistic study for TC’s anti-cancer activity is required. Thus, we elucidated the potential molecular mechanism of TC’s anti-proliferative effect and the induction of apoptosis in human colorectal cancer cells.

**Methods:**

How water extracts form TC (TC-HW) was used in this study. Anti-cell proliferative effect of TC-HW was evaluated by MTT assay. The change of protein or mRNA level by TC-HW was evaluated by Western blot and RT-RCR, respectively. The promoter construct for ATF3, NF-κB, TOP-FLASH or FOP-FLASH was used for the investigation of the transcriptional activity for ATF3, NF-κB or Wnt. siRNA for ATF3 or p65 was used for the knockdown of ATF3 and p65.

**Results:**

TC-HW reduced the cell viability in human colorectal cancer cells. TC-HW decreased cyclin D1 protein level through cyclin D1 degradation via GSK3β-dependent threonine-286 (T286) phosphorylation of cyclin D1, indicating that cyclin D1 degradation may contribute to TC-HW-mediated decrease of cyclin D1 protein level. TC-HW downregulated the expression of cyclin D1 mRNA level and inhibited Wnt activation through the downregulation of β-catenin and TCF4 expression, indicating that inhibition of cyclin D1 transcription may also result in TC-HW-mediated decrease of cyclin D1 protein level. In addition, TC-HW was observed to induce apoptosis through ROS-dependent DNA damage. TC-HW-induced ROS increased NF-κB and ATF3 activation, and inhibition of NF-κB and ATF3 activation attenuated TC-HW-mediated apoptosis.

**Conclusions:**

Our results suggest that TC-HW may suppress cell proliferation through the downregulation of cyclin D1 via proteasomal degradation and transcriptional inhibition, and may induce apoptosis through ROS-dependent NF-κB and ATF3 activation. These effects of TC-HW may contribute to the reduction of cell viability in human colorectal cancer cells. From these findings, TC-HW has potential to be a candidate for the development of chemoprevention or therapeutic agents for human colorectal cancer.

## Background

Among a verity of cancers, human colorectal cancer (CRC) has been regarded as an one of the most common types of cancer and a major cause of cancer-related mortality [1]. Although the improvements of CRC treatment have been achieved, the death rate by CRC still remains high because of the advanced, metastatic or recurrent CRC [2–4]. Therefore, the development of the novel treatment for CRC has been required.

Natural products have been long regarded as one of the potential materials for developing the anti-cancer agents. Traditional Chinese medicine (TCM) has been used for the treatment of human diseases [5, 6]. *Cinnamomum cassia* (*C. cassia*) as an aromatic plant from Lauraceae family found in southern China, Vietnam, Myanmar and Laos has been widely used for treating blood circulation disturbances, dyspepsia, allergic disease, gastritis, diabetes, and other inflammatory diseases [7]. Among the bark and twigs of *C. cassia*, the bark of *C. cassia* has been applied to treating cold intolerance, weakness, soreness and coldness of lower back and knees [8]. The bark of *C. cassia* has been reported to have neuro-protective effect, anti-inflammatory effect and anti-cancer activity [9–11]. The twigs of *C. cassia* have been widely treated for menstrual pain, fever, hypertension, diabetes and cancer [12–14]. According to the many literatures, twigs of *C. cassia* (TC) exert the pharmacological activities such as anti-allergy, insecticidal, antimicrobial, antiulcer, anti-inflammatory, vasodilatory, immune-suppressive, and neuronal death prevention, tyrosinase inhibition and anticancer, antioxidant and free radical scavenging, as well as antidiabetic and aldose reductase inhibition activities [15]. In anticancer activity, TC suppressed the abnormal proliferation in JB6 P+ cells through c-Fos degradation. However, additional molecular mechanism for the anticancer activity of TC still remains to be elucidated. In this study, we elucidated anti-cancer activity and potential molecular mechanism of TC against human colorectal cancer cells. We here reported the additional mechanism of hot-water extracts from the twigs of *Cinnamomum cassia* (TC-HW) for anti-cancer activity. TC-HW suppressed the proliferation of human colorectal cancer cells through GSK3β-dependent cyclin D1 degradation and induced ROS-dependent apoptosis in human colorectal cancer cells.

## Methods

### Materials

Dulbecco’s Modified Eagle medium (DMEM)/F-12 1:1 Modified medium (DMEM/F-12) for the cell culture was purchased from Lonza (Walkersville, MD, USA). LiCl, MG132 and 3-(4,5-dimethylthizaol-2-yl)-2,5-diphenyl tetrazolium bromide (MTT) and N-acetyl-L-cysteine (NAC) were purchased from Sigma Aldrich (St. Louis, MO, USA). Antibodies against cyclin D1, phospho-cyclin D1 (Thr286), HA-tag, β-catenin, TCF4, cleaved PARP, phospho-H2AX, IκB-α, p65 and β-actin were purchased from Cell Signaling (Bervely, MA, USA). Antibody for activating transcription factor (ATF3) was purchased from Santa Cruz Inc. (Santa Cruz, CA, USA). All chemicals were purchased from Fisher Scientific, unless otherwise specified.

### Sample preparation

The twigs of *Cinnamomum cassia* (TC) (voucher number: Jeong1001(AHN)) was purchased from Humanherb, Korea and formally identified by Jin Suk Koo as the professor of Andong National University, Korea. Twenty gram of TC was extracted with 300 ml of DH_2_O with boiling at 100 °C for 1 h. After 1 h, the hot water extracts were filtered and then freeze-dried. The hot water extracts from TC (TC-HW) was kept in a refrigerator until use.

### Cell culture and treatment

Human colorectal cancer cell lines such as HCT116, SW480, LoVo and HT-29 were purchased from Korean Cell Line Bank (Seoul, Korea) and grown in DMEM/F-12 supplemented with 10% fatal bovine serum (FBS), 100 U/ml penicillin and 100 μg/ml streptomycin. The cells were maintained at 37 °C under a humidified atmosphere of 5% CO_2_. TC-HW was dissolved in dimethyl sulfoxide (DMSO) and treated to cells. DMSO was used as a vehicle and the final DMSO concentration did not exceed 0.1% (*v*/v).

### Cell viability assay

Cell viability was evaluated by MTT assay. Briefly, cells were plated at a density of 3 × 10^4^ cells/well in 96-well plate and incubated for 24 h. The cells were treated with TC-HW at the indicated concentrations for 24 h. Then, the cells were incubated with 50 μl of MTT solution (1 mg/ml) for an additional 2 h. The resulting crystals were dissolved in DMSO. The formation of formazan was measured by reading absorbance at a wavelength of 570 nm using UV/Visible spectrophotometer (Human Cop., Xma-3000PC, Seoul, Korea).

### Measurement of intracellular ROS

Measurement of intracellular ROS was performed using OxiSelect™ Intracellular ROS Assay Kit (Cell Biolabs, Inc., San Diego, CA). Briefly, HCT116 and SW480 cells were plated at a density of 3 × 10^4^ cells/well in 96-well plate and incubated for 24 h. The cells were treated with TC-HW at the indicated concentrations for 24 h and then washed with 1 × phosphate-buffered saline (PBS). Then, the cells were stained with 100 μl of 1× dichlorofluorescein diacetate (DCFH-DA)/media solution at 37 °C for 1 h. After three washing with 1× PBS, the cells were lysed with 100 μl of 2 × cell lysis buffer, incubated for 5 min and then the fluorescence was recorded at 480 nm excitation/530 nm emission in an enzyme-linked immunosorbent assay plate reader (Human Cop., Xma-3000PC, Seoul, Korea).

### Cell cycle analysis

HCT116 cells were plated at a density of 1 × 10^6^ cells/well in 6-well plate and incubated for 24 h. The cells were treated with TC-HW for 24 h. After then, the cells were dissociated with trypsin, washed in cold PBS and fixed with 70% cold ethanol on ice for 30 min. The suspensions were centrifuged at 1500 rpm for 5 min. The pellets were resuspended in a solution containing 50 μg/ml propidium iodide, 1 mg/ml sodium citrate, 0.3 ml nonidet P-40 and 5 μg/ml RNase A and stayed on ice at least 40 min. Then the pellets were analyzed by a flow cytometer.

### Isolation of cytosol and nucleus fraction

Cytosol and nuclear fractions of cells were prepared using a nuclear extract kit (Active Motif, Carlsbad, CA, USA) according to the manufacturer’s protocols. Briefly, HCT116 cells after TC-HW treatment were harvested with 1 × cold hypotonic buffer and incubated at 4 °C for 15 min. After adding detergent and vortexing for 10 s, the cells were centrifuged at 14,000 g for 1 min at 4 °C and the supernatants (cytoplasmic fraction) were collected and stored at − 80 °C for further analysis. The cell pellets were used for nuclear fraction collection. Cell pellets were re-suspended with complete lysis buffer by pipetting up and down, and incubated at 4 °C for 30 min under shaking. After 30 min, nuclear suspensions were centrifuged at 14,000 g for 10 min at 4 °C, and the supernatants (nuclear fraction) were stored at − 80 °C for further analysis.

### SDS-PAGE and western blot

Cells were plated at a density of 2 × 10^6^ cells/well in 6-well plate and grown to 80% confluence. After treatment, the cells were washed with 1 × phosphate-buffered saline (PBS), and lysed in radioimmunoprecipitation assay (RIPA) buffer (Boston Bio Products, Ashland, MA, USA) supplemented with protease inhibitor cocktail (Sigma-Aldrich) and phosphatase inhibitor cocktail (Sigma-Aldrich), and centrifuged at 15,000 × rpm for 10 min at 4 °C. Protein concentration was determined by the bicinchoninic acid (BCA) protein assay (Pierce, Rockford, IL, USA). The proteins were separated on SDS-PAGE and transferred to PVDF membrane (Bio-Rad Laboratories, Inc., Hercules, CA, USA). The membranes were blocked for non-specific binding with 5% non-fat dry milk in Tris-buffered saline containing 0.05% Tween 20 (TBS-T) for 1 h at room temperature and then incubated with specific primary antibodies in 5% non-fat dry milk at 4 °C overnight. After three washes with TBS-T, the blots were incubated with horse radish peroxidase (HRP)-conjugated immunoglobulin G (IgG) for 1 h at room temperature and chemiluminescence was detected with ECL Western blotting substrate (Amersham Biosciences, Piscataway, NJ, USA) and visualized in Polaroid film.

### Reverse transcriptase-polymerase chain reaction (RT-PCR)

After treatment, total RNA was prepared using a RNeasy Mini Kit (Qiagen, Valencia, CA, USA) and total RNA (1 μg) was reverse-transcribed using a Verso cDNA Kit (Thermo Scientific, Pittsburgh, PA, USA) according to the manufacturer’s protocol for cDNA synthesis. PCR was carried out using PCR Master Mix Kit (Promega, Madison, WI, USA) with human primers for cyclin D1, β-catenin, TCF4, ATF3 and GAPDH as followed: cyclin D1: forward 5′-aactacctggaccgcttcct-3′ and reverse 5′-ccacttgagcttgttcacca-3′, β-catenin: forward 5′-cccactaatgtccagcgttt-3′ and reverse 5′-aatccactggtgaaccaagc-3′, TCF4: forward 5′-ttcaaagacgacggcgaacag-3′ and reverse 5′-ttgctgtacgtgataagaggcg-3′, ATF3: forward 5′-gtttgaggattttgctaacctgac-3′, and reverse 5′-agctgcaatcttatttctttctcgt-3′, GAPDH: forward 5′-acccagaagactgtggatgg-3′ and reverse 5′-ttctagacggcaggtcaggt-3′. The following PCR reaction conditions were used: 1 cycle of (3 min at 94 °C for denaturation), 25 cycles of (30 s at 94 °C for denaturation, 30 s at 60 °C for annealing, and 30 s at 72 °C for elongation), and 1 cycle of (5 min for extension at 72 °C).

### Transfection of small interference RNA (siRNA)

HCT116 cells were seeded and incubated overnight. HCT116 cells were transfected with control siRNA, p65 siRNA or ATF3 siRNA for 48 h at a concentration of 100 nM using TransIT-TKO transfection reagent (Mirus, Madison, WI, USA) according to the manufacturer’s instruction. Then HCT116 cells were treated with TC-HW (100 μg/ml) for 24 h.

### Expression vectors

HA-tagged wild type cyclin D1 and HA-tagged T286A cyclin D1 were provided from Addgene (Cambridge, MA, USA). Transient transfection of the vectors was performed using the PolyJet DNA transfection reagent (SignaGen Laboratories, Ijamsville, MD, USA) according to the manufacturers’ instruction.

### Transient transfection and luciferase activity

Transient transfection for luciferase activity was carried out using the PolyJet DNA transfection reagent (SignaGen Laboratories, Ijamsville, MD, USA) according to the manufacturers’ instruction. Cells were seeded in 12-well plates at a concentration of 2 × 10^5^ cells/well. After growth overnight, plasmid mixtures containing 1 μg of the luciferase constructs (Addgene, Cambridge, MA, USA) and 0.1 μg of pRL-null vector were transfected for 24 h. The transfected cells were treated with TC-HW for 24 h. The cells were then harvested in 1 × luciferase lysis buffer, and luciferase activity was normalized to the pRL-null luciferase activity using a dual-luciferase assay kit (Promega, Madison, WI, USA).

### GC/MS analysis of the active chemical compounds

GC–MS analysis was performed using same GC–MSD, equipped with a Ultra-2 (Crosslinked 5% PH ME Siloxane, 25 m length × 0.20 mm i.d. Hewlett packard, USA). The carrier gas used was helium, at a constant flow rate of 1.0 ml/min. One microliter of the extract was injected into the column using 10:1 of the split ratio injection mode. The oven temperature was initially held at 100 °C for 0 min, then raised to 280 °C at a rate of 3 °C/min for 50 min, and finally held at 280 °C for 5 min. The temperatures of injector and detector were 200 °C and 240 °C, respectively. The mass detector was operated in electron impact mode with an ionization energy of 70 eV, a scanning range of 33–550 a.m.u. and a scan rate of 1.4 scans/s. Components of the extracts were identified with the aid of the Wiley 275 Imass spectral database (Hewlett-Packard, 1995) or by manual interpretation.

### Statistical analysis

All the data are shown as mean ± SEM (standard error of mean). Statistical analysis was performed with one-way ANOVA followed by Dunnett’s test. Differences with *P < 0.05 were considered statistically significant.

## Results

### Effect of TC-HW on the cell viability and cyclin D1 protein level in HCT116 and SW480 cells

**Fig. 1:**
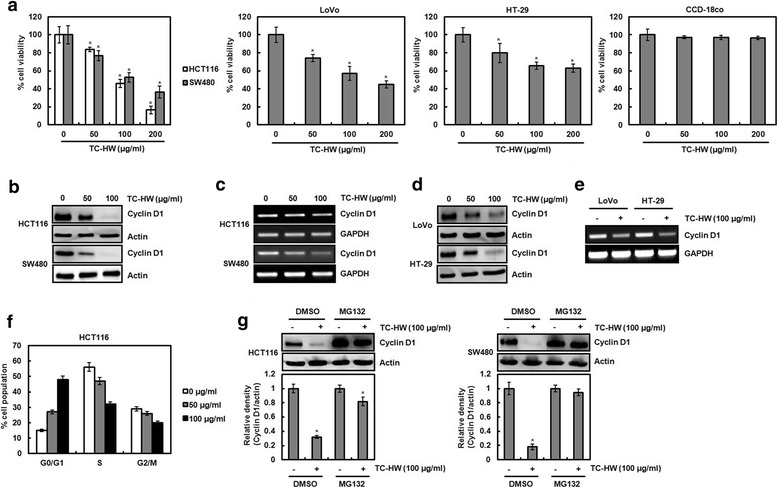
The effect of TC-HW on the cell viability and cyclin D1 expression in human colorectal cancer cells. **a** HCT116, SW480, LoVo and HT-29 cells were plated overnight and then treated with TC-HW at the indicated concentrations for 24 h. Cell viability was measured using MTT assay. *P < 0.05 compared to cell without TC-HW. **b-e** HCT116, SW480, LoVo and HT-29 cells were plated overnight and then treated with TC-HW at the indicated concentrations for 24 h. For Western blot analysis (**b**, **d**), cell lysates were subjected to SDS-PAGE and the Western blot was performed using antibody against cyclin D1. Actin was used as internal control for Western blot analysis. For RT-PCR analysis of the gene expression of cyclin D1 (**c**, **e**), total RNA was prepared. GAPDH was used as internal control for RP-PCR. **f** HCT116 cells were plated overnight and then treated with TC-HW at the indicated concentrations for 24 h. Cell cycle progression was analyzed by flow cytometer. **g** HCT116 and SW480 cells were pretreated with MG132 (10 μM), and then co-treated with TC-HW (100 μg/ml). Cell lysates were subjected to SDS-PAGE and the Western blot was performed using antibody against cyclin D1. Actin was used as internal control for Western blot analysis. *P < 0.05 compared to cell without TC-HW

To evaluate whether TC-HW reduces cell viability in human colorectal cancer cell lines, HCT116, SW480, LoVo and HT-29 cells, MTT assay was performed. As shown in Fig. 1a, TC-HW reduced the cell viability by 16.3% and 23.0% at 50 μg/ml, 54.0% and 47.4% at 100 μg/ml, and 83.5% and 63.6% at 200 μg/ml in HCT116 and SW480 cells, respectively. In addition, we observed that the viability of LoVo and HT-29 cells was dose-dependently reduced by TC-HW treatment. However, TC-HW did not affect the cell viability in the normal cells (Fig. 1a). Because cell growth arrest contributes to the decrease of the cell viability, we investigated whether TC-HW modulates cyclin D1 protein level in HCT116 and SW480 cells because cyclin D1 has been regarded as one of the proteins regulating the cell proliferation. As a result, cyclin D1 protein level was decreased by TC-HW treatment at the dose-dependent manner in HCT116 and SW480 cells (Fig. 1b). Because cyclin D1 protein level is regulated by gene amplification [16], we investigated whether TC-HW affects cyclin D1 transcription. As shown in Fig. 1c, TC-HW treatment downregulated the expression of cyclin D1 mRNA. TC-HW-mediated downregulation of cyclin D1 at the protein and mRNA level was observed in LoVo and HT-29 cells (Fig. 1d and e). We investigated whether the TC-HW-mediated downregulation of cyclin D1 contributes to the cell cycle arrest. As shown in Fig. 1f, the majority of HCT116 cells without TC-HW were in S phase. However, TC-HW dose-dependently induced the accumulation of G0/G1 phase in HCT116 cells. We observed that the downregulation of cyclin D1 protein level by TC-HW is more dramatically showed than the change of cyclin D1 mRNA level, which indicates that TC-HW may affect cyclin D1 protein stability. To determine whether TC-HW may induce cyclin D1 proteasomal degradation, HCT116 and SW480 cells were pretreated with MG132 as a proteasome inhibitor and then co-treated with TC-HW. As shown in Fig. 1g, cyclin D1 protein level was decreased by TC-HW in absence of MG132. However, the presence of MG132 restored TC-HW-mediated cyclin D1 downregulation. These data suggest that TC-HW-mediated downregulation of cyclin D1 may result from both inhibition of cyclin D1 transcription and induction of cyclin D1 proteasomal degradation.

### TC-HW mediated cyclin D1 degradation is dependent on GSK3β-dependent threonine-286 (T286) of cyclin D1

**Fig. 2:**
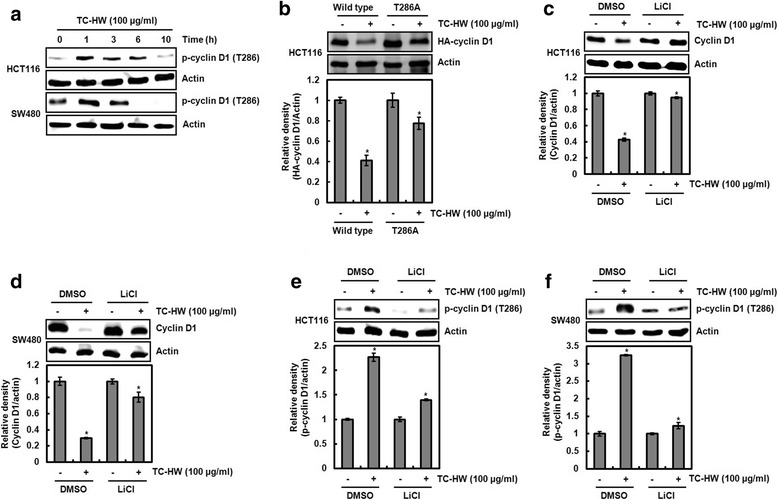
TC-HW-mediated cyclin D1 degradation dependent on GSK3β-mediated Thr-286 phosphorylation of cyclin D1. **a** HCT116 and SW480 were plated overnight and then treated with TC-HW (100 μg/ml) for the indicated times. Cell lysates were subjected to SDS-PAGE and the Western blot was performed using antibody against phospho-cyclin D1 (Thr-286). Actin was used as internal control for Western blot analysis. **b** HCT116 cells were transfected with wild type HA-tagged cyclin D1 or HA-tagged T286A cyclin D1 expression vector and then treated with 100 μg/ml of TC-HW. Cell lysates were subjected to SDS-PAGE and the Western blot was performed using antibody against HA-cyclin D1. Actin was used as internal control for Western blot analysis. *P < 0.05 compared to cell without TC-HW. **c** and **d** HCT116 and SW480 cells were pretreated with LiCl (20 mM), and then co-treated with TC-HW (100 μg/ml). Cell lysates were subjected to SDS-PAGE and the Western blot was performed using antibody against cyclin D1. Actin was used as internal control for Western blot analysis. *P < 0.05 compared to cell without TC-HW. **e** and **f** HCT116 and SW480 cells were pretreated with LiCl (20 mM), and then co-treated with TC-HW (100 μg/ml). Cell lysates were subjected to SDS-PAGE and the Western blot was performed using antibody against phospho-cyclin D1 (Thr-286). Actin was used as internal control for Western blot analysis. *P < 0.05 compared to cell without TC-HW

There is growing evidence that cyclin D1 degradation is a consequence of T286 phosphorylation of cyclin D1 protein [17]. Thus, we firstly investigated whether TC induces T286 phosphorylation of cyclin D1. As shown in Fig. 2a, TC-HW phosphorylated cyclin D1 T286 at 1 h after the treatment. Next, we determined that the mutation of cyclin D1 T286 affects TC-HW-mediated cyclin D1 degradation. As a result, cyclin D1 was degraded by TC-HW in the cells transfected with wild-type cyclin D1, while TC-HW-mediated cyclin D1 degradation was attenuated in the cells transfected with T286A-cyclin D1 (Fig. 2b). These data indicate that cyclin D1 degradation by TC-HW may be a consequence of T286 phosphorylation of cyclin D1.

GSK3β-dependent T286 phosphorylation of cyclin D1 has been known to be involved in cyclin D1 degradation [17–19]. Thus, LiCl as a GSK3β inhibitor was applied for determining whether TC-HW-mediated cyclin D1 degradation is GSK3β-dependent. As shown in Fig. 2c and d, TC-HW degraded cyclin D1 protein in absence of LiCl, while cyclin D1 degradation by TC-HW was attenuated in presence of LiCl. In addition, we observed that the inhibition of GSK3β by LiCl attenuated T286 phosphorylation of cyclin D1 induced by TC-HW in HCT116 and SW480 cells (Fig. 2e and f). These data indicate that TC-HW may induce GSK3β-dependent T286 phosphorylation of cyclin D1 and subsequently degrade cyclin D1 protein.

Although ERK1/2 and p38 has been reported to be associated with cyclin D1 degradation [20, 21], TC-HW induced cyclin D1 degradation in absence/presence of PD98059 as an ERK1/2 inhibitor and SB203580 as a p38 inhibitor (data not shown).

### TC-HW inhibits Wnt activation

**Fig. 3:**
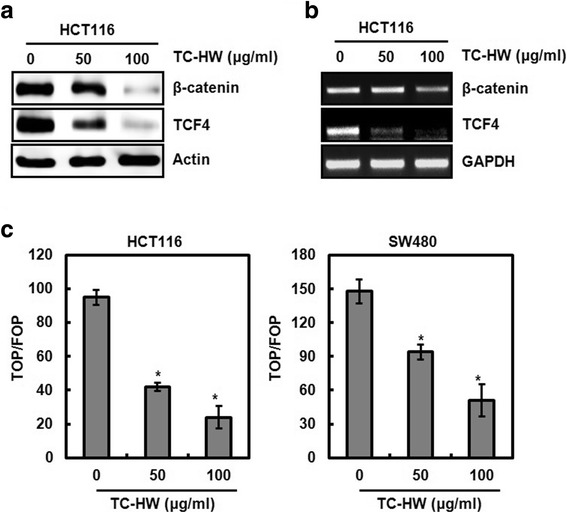
Inhibition of cyclin D1 transcription by TC-HW through the inhibition of Wnt activation. **a** and **b** HCT116 and SW480 cells were treated with TC-HW at the indicated concentrations for 24 h. For Western blot analysis (**a**), cell lysates were subjected to SDS-PAGE and the Western blot was performed using antibodies against β-catenin or TCF4. Actin was used as internal control for Western blot analysis. For RT-PCR analysis of the gene expression of β-catenin and TCF4 (**b**), total RNA was prepared. GAPDH was used as internal control for RP-PCR. **c** HCT116 and SW480 cells were co-transfected with TOP-FLASH or FOP-FLASH constructs containing wild-type or mutated TCF binding sites and pRL-null. The cells were treated with TC-HW for 24 h. Luciferase activity for TOP-FLASH and FOP-FLASH was measured as a ratio of firefly luciferase signal/renilla luciferase signal using a dual luciferase assay kit. *P < 0.05 compared to cell without TC-HW

In this study, we observed that TC-HW suppresses cyclin D1 transcriptional activity (Fig. 1c). Because Wnt signaling has been reported to be associated with cyclin D1 transcription [22], we investigated whether TC-HW regulates Wnt activation. As a result, TC-HW treatment resulted in the decrease of β-catenin and TCF4 in both protein and mRNA level (Fig. 3a and b), which contributed to the inhibition of β-catenin/TCF-dependent luciferase activity (Fig. 3c). These data indicate that TC-HW may suppress cyclin D1 transcription through inhibiting Wnt activation via downregulation of β-catenin and TCF4.

### TC-HW induces ROS-dependent apoptosis

**Fig. 4:**
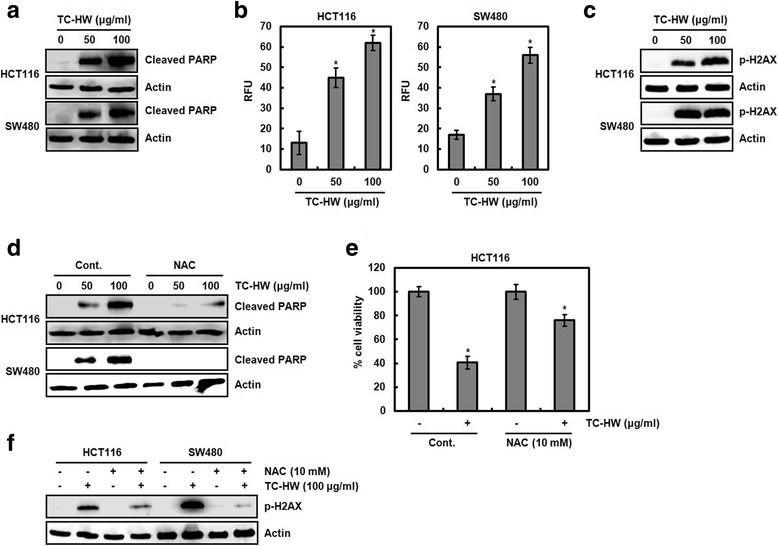
Induction of apoptosis by TC-HW through ROS-dependent DNA damage. **a** HCT116 and SW480 were plated overnight and then treated with TC-HW at the indicated concentrations for 24 h. Cell lysates were subjected to SDS-PAGE and the Western blot was performed using antibody against cleaved PARP. Actin was used as internal control for Western blot analysis. **b** HCT116 and SW480 cells were plated overnight and then treated with TC-HW at the indicated concentrations for 24 h. Intracellular ROS were measured using OxiSelect™ Intracellular ROS Assay Kit. *P < 0.05 compared to cell without TC-HW. **c** HCT116 and SW480 were plated overnight and then treated with TC-HW at the indicated concentrations for 24 h. Cell lysates were subjected to SDS-PAGE and the Western blot was performed using antibody against phospho-H2AX. Actin was used as internal control for Western blot analysis. **d** HCT116 and SW480 were pretreated with NAC (10 mM) and then co-treated with TC-HW at the indicated concentrations. Cell lysates were subjected to SDS-PAGE and the Western blot was performed using antibody against cleaved PARP. Actin was used as internal control for Western blot analysis. **e** HCT116 and SW480 were pretreated with NAC (10 mM) and then co-treated with TC-HW (100 μg/ml). Cell viability was measured using MTT assay. *P < 0.05 compared to cell without TC-HW. **f** HCT116 and SW480 were pretreated with NAC (10 mM) and then co-treated with TC-HW (100 μg/ml). Cell lysates were subjected to SDS-PAGE and the Western blot was performed using antibody against phospho-H2AX. Actin was used as internal control for Western blot analysis

Apoptosis as well as the cell growth arrest has been involved in the reduction of the cell viability. Thus, we determined whether TC-HW induces apoptosis in human colorectal cancer cells. As shown in Fig. 4a, cleavage of PARP as an apoptotic marker was increased in TC-HW-treated HCT116 and SW480 cells at the dose-dependent manner. In addition, we observed that TC-HW treatment increases the intracellular ROS level in HCT116 and SW480 cells (Fig. 4b).

There is growing evidence that ROS-mediated apoptosis is associated with DNA damage [23–26]. Thus, the effect of TC-HW on DNA damage was investigated, and we observed that TC-HW increases the phosphorylation of H2AX as a DNA damage marker (Fig. 4c). These data suggest the hypothesis that TC-HW may reduce the cell viability through inducing apoptosis via ROS-mediated DNA damage. To elucidate this hypothesis, we investigated the effect of TC-HW on PARP cleavage, cell viability and phosphorylation of H2AX under ROS scavenging by N-acetyl-l-cysteine (NAC). As shown in Fig. 4d-f, the presence of NAC attenuated the cleavage of PARP, reduction of the cell viability, and phosphorylation of H2AX mediated by TC-HW.

### ROS-dependent activation of NF-κB contributes apoptosis

**Fig. 5:**
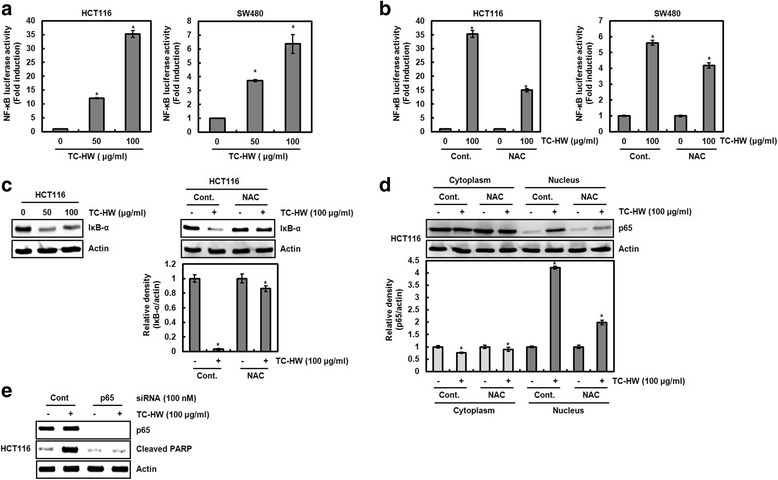
Contribution of ROS-dependent NF-κB activation to TC-HW-mediated apoptosis. **a** and **b**HCT116 and SW480 were co-transfected with NF-κB luciferase construct and pRL-null. The cells were treated with TC-HW in presence/absence of NAC. Luciferase activity for NF-κB was measured as a ratio of firefly luciferase signal/renilla luciferase signal using a dual luciferase assay kit. *P < 0.05 compared to cell without TC-HW. **c** HCT116 cells were treated with TC-HW (100 μg/ml) in presence/absence of NAC. Cell lysates were subjected to SDS-PAGE and the Western blot was performed using antibody against IκB-α. Actin was used as internal control for Western blot analysis. *P < 0.05 compared to cell without TC-HW. **d** HCT116 cells were treated with TC-HW (100 μg/ml) in presence/absence of NAC. Cytosol and nucleus extracts were prepared and subsequently Western blot analysis was performed for p65. Actin was used as internal control for Western blot analysis. *P < 0.05 compared to cell without TC-HW. **e** HCT116 cells were transfected with control and p65 siRNA and then treated with TC-HW (100 μg/ml). Cell lysates were subjected to SDS-PAGE and the Western blot was performed using antibody against p65 or cleaved PARP. Actin was used as internal control for Western blot analysis

ROS-mediated NF-κB activation was reported to induce apoptosis in human colorectal cancer cells [27–29]. Thus, we investigated the relationship between TC-HW-mediated ROS, NF-κB activation, and apoptosis. Firstly, we observed that TC-HW dose-dependently increases NF-κB luciferase activity (Fig. 5a), while NAC treatment blocks NF-κB activation by TC-HW (Fig. 5b). NF-κB activation is regulated by p65 nuclear accumulation through IκB-α degradation [30]. As shown in Fig. 5c and d, IκB-α degradation and subsequent p65 nuclear accumulation by TC-HW treatment were attenuated in presence of NAC. These data indicate that TC-HW-mediated ROS may contribute to NF-κB activation. Lastly, we determined whether NF-κB activation is essential for the TC-HW-mediated apoptosis. As shown in Fig. 5e, p65 knockdown by p65 siRNA decreased TC-HW-mediated cleavage of PARP compared to the cell transfected with control-siRNA.

### ROS-dependent activation of activating transcription factor 3 (ATF3) contributes to apoptosis

**Fig. 6:**
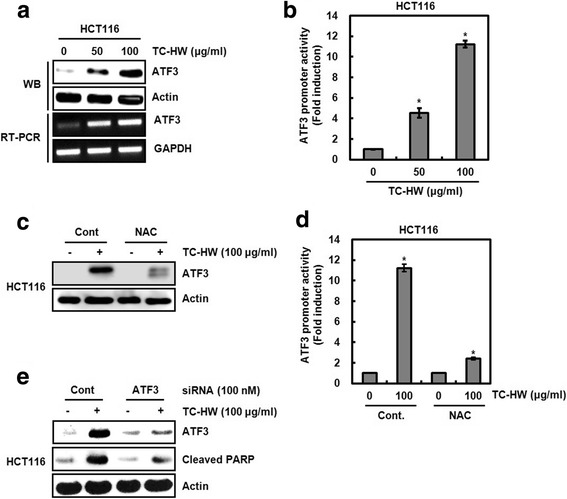
Contribution of ROS-dependent ATF3 activation to TC-HW-mediated apoptosis. **a** and **b** HCT116 and SW480 cells were treated with TC-HW at the indicated concentrations for 24 h. For Western blot analysis, cell lysates were subjected to SDS-PAGE and the Western blot was performed using antibodies against ATF3. Actin was used as internal control for Western blot analysis. For RT-PCR analysis of the gene expression of ATF3, total RNA was prepared. GAPDH was used as internal control for RP-PCR. **b** HCT116 cells were co-transfected with ATF3 promoter and pRL-null for 24 h, and then treated with TC-HW at the indicated concentrations for 24 h. Luciferase activity for ATF3 promoter activity was measured as a ratio of firefly luciferase signal/renilla luciferase signal using a dual luciferase assay kit. *P < 0.05 compared to cell without TC-HW. **c** HCT116 cells were treated with TC-HW (100 μg/ml) in presence/absence of NAC. Cell lysates were subjected to SDS-PAGE and the Western blot was performed using antibody against ATF3. Actin was used as internal control for Western blot analysis. **d** HCT116 cells were co-transfected with ATF3 promoter and pRL-null for 24 h, and then treated with TC-HW (100 μg/ml) in presence/absence of NAC. Luciferase activity for ATF3 promoter activity was measured as a ratio of firefly luciferase signal/renilla luciferase signal using a dual luciferase assay kit. *P < 0.05 compared to cell without TC-HW. **e** HCT116 cells were transfected with control and ATF3 siRNA and then treated with TC-HW (100 μg/ml). Cell lysates were subjected to SDS-PAGE and the Western blot was performed using antibody against ATF3 or cleaved PARP. Actin was used as internal control for Western blot analysis

It has been reported that activating transcription factor 3 (ATF3) is one of the target proteins for ROS and ROS-mediated ATF3 activation induces apoptosis in human colorectal cancer cells [27]. Thus, we firstly tested whether TC-HW affects ATF3 expression. As shown in Fig. 6a, we observed that TC-HW dose-dependently increases ATF3 in both protein and mRNA level, indicating that TC-HW-mediated overexpression of ATF3 may result from ATF3 transcriptional regulation. In the experiment for TC-HW-mediated ATF3 transcriptional activation, ATF3 promoter activity was dramatically increased by TC-HW treatment (Fig. 6b). Secondly, we determined that TC-HW-mediated ROS affects ATF3 expression and TC-HW-mediated increase of ATF3 protein level and ATF3 promoter activity were decreased in presence of NAC (Fig. 6c and d). Lastly, we investigated that TC-HW-mediated ATF3 expression contributes to the induction of apoptosis, and observed that ATF3 knockdown by ATF3 siRNA blocks TC-HW-mediated cleavage of PARP (Fig. 6e).

### GC/MS analysis of the active chemical compounds

**Fig. 7:**
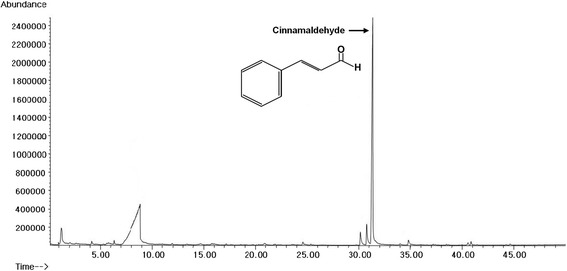
The chromatography of GC/MS analysis of TC-HW

We analyzed the potential medicinal components with anti-cancer properties from TC-HW using GC/MS. As shown in Fig. 7, cinnamaldehyde (C6H5CH=CHCHO, MW: 132.16) were identified. Indeed, cinnamaldehyde has been reported to have anti-cancer properties. In the quantitative analysis, 15.4% of cinnamaldehyde was contained in TC-HW used in this study.

## Discussion

Abnormal cell proliferation induced by altered expression of the protein related with the cell cycle is an important part of cancer development and progression [31]. Thus, the regulation of the cell proliferation has been regarded as an one of the anticancer targets and many anticancer agents have been reported to kill the cancer cells through blocking cancer cell proliferation vis cell cycle arrest [31]. Cyclin-CDK complex has been known to be one of the regulators associated with the cell cycle progression [32]. Among the cyclin types, cyclin D1 has been observed to be overexpressed in human cancer and cyclin D1-CDK4/6 complex induces the phosphorylation of the retinoblastoma protein, resulting to inducing G1/S transition [16]. Thus, cyclin D1 has been suggested to be one of the important targets for the anti-cancer drug development [16]. Indeed, cyclin D1 overexpression occurs in one-third or more of human colorectal cancers [33].

In this study, we observed that TC-HW downregulates cyclin D1 at both protein and mRNA level in human colorectal cancer cells. For the elucidation of the signaling associated with TC-HW-mediated inhibition of cyclin D1 transcription, we observed that TC-HW suppresses Wnt activation through the downregulation of β-catenin and TCF4 associated with cyclin D1 gene amplification [22].

In the regulatory effect of TC-HW on cyclin D1 expression, we observed that TC-HW-mediated decrease of cyclin D1 protein level is more dramatic than the change of cyclin D1 mRNA level. These data indicate that cyclin D1 protein stability as well as cyclin D1 transcriptional inhibition may contribute to the downregulation of cyclin D1 protein level. Indeed, there is growing evidence that increased mRNA and protein level account for 2.5% and 55% in human colorectal cancer, respectively, which indicates that the upregulated cyclin D1 protein level does not occur solely as a consequence of gene amplification [16] and the defective post-translational regulation such as the proteasomal degradation may result in the upregulation of cyclin D1 protein level [34, 35]. Thus, induction of cyclin D1 degradation has been regarded as one of the potential anti-cancer targets [17]. In this study, it was observed that the presence of MG132 as a proteasome inhibitor attenuated TC-HW-mediated decrease of cyclin D1 protein level in HCT116 and SW480 cells, which indicates that TC-HW may induce cyclin D1 degradation.

Cyclin D1 degradation has been reported to be followed by the phosphorylation of cyclin D1 threonine-286 (T286) [19, 36]. Defective cyclin D1 degradation by the mutation of Thr-286 contributes to the upregulation of cyclin D1 protein level in several cancers, which results in the significant increase of cyclin D1’s oncogenic potential. [17, 37]. Thus, we investigated whether T286 phosphorylation affects TC-HW-mediated cyclin D1 degradation. In this study, TC-HW treatment was observed to phosphorylate T286 of cyclin D1. In addition, the mutation of threonine-286 to alanine (T286A) blocked cyclin D1 degradation by TC-HW. These data suggest that T286 phosphorylation may be an important event for TC-HW-mediated cyclin D1 degradation.

There is growing evidence that glycogen synthase kinase 3β (GSK3β) phosphorylates T286 of cyclin D1 and inhibition of GSK3β activity reduces cyclin D1 degradation [17, 19, 38]. In this study, we observed that inhibition of GSK3β by LiCl reduces TC-HW-mediated cyclin D1 degradation in HCT116 and SW480. In addition, T286 phosphorylation of cyclin D1 by TC-HW was attenuated in presence of LiCl as a GSK3β inhibitor. Our data indicates that TC-HW-mediated cyclin D1 degradation may be attributed to GSK3β-dependent T286 phosphorylation of cyclin D1.

The inactivation of apoptosis is central to the development of cancer [39] and little apoptosis has been regarded as one of the scenarios in human cancer, which results in malignant cells [40]. Therefore, apoptosis has been used for the cancer treatment of many anticancer agents [40]. Although apoptosis can be regulated by many complex pathways, reactive oxygen species (ROS) have been known to be one of many factors regulating apoptosis [41]. Disproportionate ROS induce DNA damage, which contributes to the induction of apoptosis in human cancer [23–26]. Thus, increase of intracellular ROS has been regarded as one of the targets for the cancer treatment [41]. In this study, TC-HW dose-dependently increased the cleavage of PARP, indicating that TC-HW may induce apoptosis in human colorectal cancer cells. In the study for the effect of ROS on TC-HW-mediated apoptosis, we observed that TC-HW increases ROS level and phosphorylates H2AX as a DNA damage marker. In addition, the presence of N-acetyl-l-cysteine (NAC) as a ROS scavenger attenuated TC-HW-mediated PARP cleavage and H2AX phosphorylation. These data indicate that TC-HW-mediated apoptosis may be a consequence of ROS-dependent DNA damage.

ROS-induced DNA damage can activate NF-κB signaling, resulting in the induction of apoptosis [27–29]. In addition, ROS increase the expression of activating transcription factor 3 (ATF3) and ROS-mediated ATF3 activation induces apoptosis in human colorectal cancer cells [27]. Therefore, we examined if TC-HW-induced ROS directly affects the activation of NF-κB and ATF3, which induces apoptosis in human colorectal cancer cell. TC-HW increased NF-κB transcriptional activity, while the presence of NAC blocked NF-κB activation by TC-HW. We observed that TC-HW-mediated NF-κB activation is followed by p65 nuclear accumulation through ROS-dependent degradation of IκB-α. In addition, inhibition of NF-κB activation by p65 siRNA attenuated TC-HW-mediated apoptosis. These findings suggest that ROS-dependent NF-κB activation may contribute to TC-HW-mediated apoptosis. In the effect of TC-HW on ATF3-mediated apoptosis, TC-HW increased ATF3 expression through upregulating ATF3 transcriptional activity. However, pretreatment of NAC reduced TC-HW-mediated increase of ATF3 protein level and ATF3 transcriptional activity. In addition, knockdown of ATF3 by ATF3 siRNA attenuated TC-HW-mediated apoptosis.

## Conclusion

**Fig. 8:**
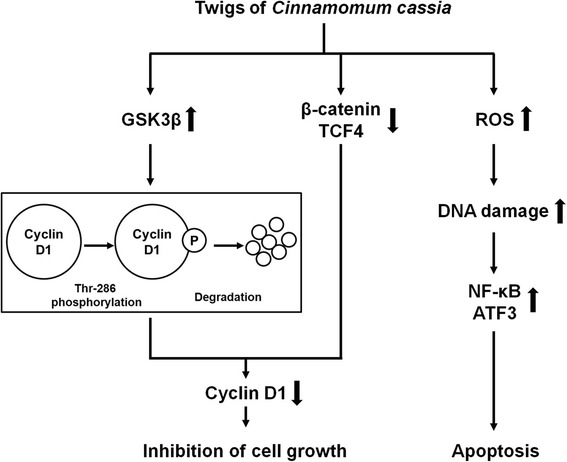
The potential mechanism of TC-HW for anticancer activity

In conclusion, TC-HW downregulates cyclin D1 protein level through cyclin D1 degradation via GSK3β-dependent T286 phosphorylation, and TC-HW-mediated inhibition of Wnt activation by the downregulation of β-catenin and TCF4 contributes to decrease of cyclin D1 protein level through the inhibition of cyclin D1 transcription (Fig. 8). In addition, TC-HW induces apoptosis through ROS-dependent activation of NF-κB and ATF3 (Fig. 8). This study may support the anti-cancer property of TC-HW and our data will provide the complementary and alternative use of TC-HW for cancer treatment.

## Abbreviations

ATF3: Activating transcription factor 3
DMSO: Dimethyl sulfoxide
ERK1/2: Extracellular signal–regulated kinase 1/2;
GSK3β: Glycogen synthase kinase 3β
IκB-α: Inhibitor of kappa B-α
MTT: 3-(4,5-dimethylthizaol-2-yl)-2,5-diphenyl tetrazolium bromide
NAC: N-acetyl-L-cysteine
NF-κB: Nuclear factor-kappa B
ROS: Reactive oxygen species
RT-PCR: Reverse transcriptase-polymerase chain reaction
SiRNA: Small interfering RNA
T286A: Threonine-286 to alanine
TC: Twigs of *Cinnamomum cassia*
TCF4: T-cell factor-4
TC-HW: Hot water extracts from twigs of *Cinnamomum cassia*
Thr286: Threonine-286
